# Stroke prevalence and associated factors among older patients with hypertension attending public healthcare facilities in Greater Kampala Metropolitan Area, Uganda

**DOI:** 10.21203/rs.3.rs-5867126/v1

**Published:** 2025-02-03

**Authors:** Bridget Nagawa Tamale, Christine Muhumuza, Aisha Nalugya, John Bosco Isunju, Richard K Mugambe, Doreen Nakalembe, Asadi Lusabe, Scovia Nalugo Mbalinda, Martin N Kaddumukasa, Tonny Ssekamatte, Christine Nalwadda Kayemba

**Affiliations:** Makerere University; Makerere University; Makerere University; Makerere University; Makerere University; Makerere University; Makerere University; Makerere University; Makerere University; Makerere University; Makerere University

**Keywords:** Stroke, Prevalence, Older patients, Hypertension, Uganda

## Abstract

**Background:**

Globally, stroke is one of the top three leading causes of death and disability. Although several stroke risk factors are modifiable, including hypertension, factors associated with stroke among older patients with hypertension in Uganda remain underexplored. This study assessed the prevalence and factors associated with stroke among older patients with hypertension in public healthcare facilities in the Greater Kampala Metropolitan Area, Uganda.

**Methods:**

A cross-sectional study was conducted among 383 older patients with hypertension. Systematic sampling was used to recruit study participants, and STATA 15.0 was used for analysis. Descriptive statistics were used to present continuous variables, while frequencies and proportions were used to present categorical data. Bivariate analyses identified associations between independent variables and stroke. Multivariable analyses controlled for confounders. A modified Poisson regression analysis with robust standard errors estimated prevalence ratios.

**Results:**

Of the 383 respondents, 71.0% (272/383) were aged 60–69 years (mean age 66.8 ± 7.1), 80.9% (310/383) were female, and 42.8% (164/383) had a primary education level (1–7 years). About 31.9% (122/383) exercised regularly, 94.8% (363/383) consumed carbohydrates frequently, 5.2% (20/383) had ever smoked, and 42.0% (151/383) had ever consumed alcohol. The prevalence of stroke was 18.3% (70/383). The factors associated with stroke included being aged 80 years and above (APR = 2.68, 95% CI: 1.59–4.51), having 8–13 years of formal education (secondary education)(APR = 0.37, 95% CI: 0.14–0.98), possessing health insurance (APR = 3.34, 95% CI: 1.19–9.37), having high knowledge of stroke (APR = 24.72, 95% CI: 6.20–98.55), and receiving stroke-related health information (APR = 1.78, 95% CI: 1.05–3.02).

**Conclusion and recommendation:**

This study demonstrated a high prevalence of stroke among older patients with hypertension. Public health education and community outreach should be expanded to underserved populations, while age-specific hypertension management and affordable healthcare services are essential. Engaging men and leveraging stroke survivors as peer educators can further strengthen prevention efforts. Future research should explore barriers to prevention and develop tailored interventions for diverse populations

## Background

Stroke is the second leading cause of death and the third leading cause of disability worldwide, following heart disease (1, 2). According to the Global Stroke Fact Sheet 2022, high systolic blood pressure stands as the single most significant risk factor for stroke, accounting for 79.6 million DALYs (67.7–90.8), or 55.5% of total stroke DALYs (48.2–62.0) worldwide (3). World Health Organisation (WHO) estimates that by 2030, 80% of all strokes will occur in people living in low and middle-income countries including Uganda, where it will account for 7.9% of all mortality (4, 5). This disproportionate burden has posed an unprecedented challenge for families with limited resources (6). Stroke rates are increasing in Sub-Saharan Africa, especially Uganda, where stroke awareness and therapeutic interventions are very limited (7). Although poorly documented, stroke is currently estimated to be the sixth highest-ranking cause of death and disability in Uganda (8).

Notably, older individuals with hypertension are at a heightened risk of stroke owing to lifestyle factors (diet, physical activity), socio-economic status, access to healthcare, and genetic predispositions. Interventions have primarily focused on hypertension management, lifestyle modifications, and access to timely medical care (9, 10). As evidenced by existing research, stroke mortality and morbidity among patients with hypertension could be considerably reduced by implementing organized stroke care, which encompasses evidence-based clinical practice guidelines, continuous quality improvement philosophy, and programs (11, 12). The current challenge lies in effectively implementing these interventions, especially in resource-scarce regions such as Uganda. In line with this, the World Stroke Organization (WSO) aims to minimize the global burden of stroke through prevention, treatment, and long-term care. In 2014, Lindsay et al. developed a Global Stroke Services Action Plan, founded on recommendations from the 10 stroke guidelines, receiving scores above 60% on two (13). This Action Plan outlines essential components of stroke care across various healthcare models (13).

Moreover, in Uganda, stroke care is guided by several policies and frameworks that aim to improve healthcare delivery. For instance, the National Multisectoral Strategic Plan for the Prevention and Control of Non-Communicable Diseases (NCDs) 2018–2023 seeks to reduce risk factors and mortality associated with NCDs, including stroke, by enhancing prevention and control measures (14). Additionally, the National Health Policy (2010–2020) and the Universal Health Coverage (UHC) Roadmap 2019 emphasize the need for equitable, accessible, and quality healthcare for all Ugandans, including stroke prevention and management. Despite the existence of these frameworks, Uganda still lacks a comprehensive, coordinated stroke care policy that integrates prevention, treatment, and long-term care. Currently, stroke management is addressed within the broader Uganda Clinical Guidelines (15), but more targeted efforts are needed to develop and implement stroke-specific policies and care pathways in the country.

While extensive studies devoted to the management and prevention of stroke, the global burden of stroke would not be reduced without efforts targeting understanding the stroke burden among high-risk groups such as older patients with hypertension (16). Therefore, this study aimed to assess the prevalence and factors associated with stroke among older patients with hypertension attending public healthcare facilities in GKMA, Uganda. It also established the practices towards stroke prevention among older patients with hypertension. Generated evidence informed the design of more precise interventions to combat, control, and prevent stroke among patients with hypertension.

## Methods

### Study setting and design

This was a cross-sectional study conducted in public healthcare facilities in GKMA that manage NCDs including hypertension. GKMA includes three districts that is, Kampala, Wakiso, and Mukono districts. According to the Uganda Bureau of Statistics Population projection 2021, the population of Kampala is 1,709,900, the population of Mukono is 720,100, and the population of Wakiso is 3,105,700 (17). Uganda’s health facilities are classified into seven levels based on the services they provide and the catchment area they are intended to serve. The health facilities are designated as Health Centre Level One (HC I) to Health Centre Level Four (HC IV); General Hospital, Regional Referral Hospital, and National Referral Hospital. In the districts of Wakiso, Mukono, and Kampala, there are 72, 40, and 26 public healthcare facilities, respectively (18). MOH mandates public healthcare facilities to prevent, manage, and control NCDs, including stroke and hypertension, through educating the community on healthy lifestyles and early detection of diseases; screening for NCDs; follow-up cases; and promoting community-based rehabilitation; and appropriate referral (19). KCCA clinics run integrated NCD clinics twice a week. In these clinics, all patients with NCDs (both HIV positive and negative) are seen. These include patients with hypertension, diabetes, and chronic lung diseases, among others. The clinics are manned by medical officers, clinical officers, and nurses. In both clinics, an approximate number of 20–40 patients are seen per clinic visit.

### Study population and eligibility criteria

This study was conducted among older patients with hypertension attending public healthcare facilities in GKMA. This study defined older patients as those aged 60 years and above. This definition is recommended by the United Nations as well as the Uganda National Plan of Action For Older Persons (20). Patient with hypertension aged 60 years and above who were receiving treatment at public healthcare facilities in the GKMA and had given their informed consent to participate were included in the study. Patients who were critically ill, incapacitated, or otherwise unable to endure the study procedures were excluded.

### Sample size estimation

The sample size was determined using the Kish Leslie formula (21). Based on an assumption of a prevalence of 33.7% of stroke among adult Ugandans in rural and urban Mukono district (22), a 5% margin of error, and a 95% confidence interval, a sample of 344 participants was achieved. After accounting for a non-response (nr) rate of 10%, we obtained a final sample size of **383** respondents.

### Sampling methods

Systematic sampling was used to select study participants. A list of 19 public healthcare facilities with NCD clinics in Kampala (6 facilities), Wakiso (8 facilities), and Mukono (5 facilities) districts was obtained from the respective District and/or Municipal Health Departments. From this list, seven (7) high-volume health facilities were selected to ensure a substantial number of older patients with hypertension were included in the study (Table 1). High-volume facilities were defined by the frequency of NCD clinic services (offered twice a week) and the number of patients with hypertension seen per clinic visit, typically ranging from 20 to 60 patients.

At each selected facility, a list of all older patients with hypertension attending the clinic on the day of data collection was compiled by the clinic officer/nurse in charge. Systematic sampling was then applied to recruit participants. The sampling interval (K) was determined by dividing the total number of older patients with hypertension present at each facility (N) by the desired sample size for that facility (n) using the formula: K = N/n. For example, if a facility had 40 older patients with hypertension and the desired sample size for that facility was 20, the sampling interval K would be K = 40/20 = 2. This meant that every 2nd patient on the list was selected. A random starting point was chosen within the first sampling interval, and every subsequent K^th^ patient was included in the sample. This process was repeated at each facility, ensuring proportional representation based on the number of older hypertensive patients at each site.

The total number of respondents for each facility was determined proportionate to the relative number of older hypertensive patients attending that facility, compared to the total number of older hypertensive patients across all facilities. The formula used was:

$$N=\frac{Number\;of\;older\;hypertensives\;in\;a\;given\;HCF}{total\;number\;of\;older\;hypertensives\;in\;all\;HCFs}\times calculated\;sample\;size$$

### Study variables and measurements

The primary outcome variable for this study is having a stroke, measured as a binary variable (Yes/No) indicating whether a participant has experienced a stroke. This was determined through self-reported data, with participants being asked the specific question, “Have you ever suffered a stroke?” Recognizing that not all participants may be familiar with the medical term "stroke," the question was operationalized to include a descriptive explanation for those who needed clarification. For participants who were uncertain or unfamiliar with the term, stroke was described in accessible terms, including symptoms such as sudden weakness or numbness in the face, arm, or leg (especially on one side of the body), difficulty speaking or understanding speech, sudden trouble seeing, walking, or loss of balance. Participants who understood the term “stroke” were asked directly, while those who required further explanation received this description to ensure accurate responses. The prevalence of stroke in the study was calculated as the proportion of patients with hypertension aged 60 years and above who reported having experienced a stroke. This method was also used by Sanuade, Dodoo (23) to assess the prevalence and correlates of stroke among older adults in Ghana. Independent variables included: 1) socio-demographic factors (such as age, sex, education level, marital status, religion, employment status, and dependents); 2) patient factors (such as patient beliefs, and attitudes regarding stroke, and patients’ knowledge on stroke ; 3) lifestyle factors (e.g., alcohol intake, diet, physical activity), 4) hypertension treatment-related factors (including drug regimen, duration of hypertensive treatment, route of medication, cost of the drug, drug adverse-related complications, number of drugs, and frequency per day among others), and 5) Health system factors (including distance to the health facility, waiting time, availability of drugs, follow-up and monitoring of patients, patient-health worker relationship, and availability of insurance services).

Patients’ knowledge of stroke was adapted from a study by Woldetsadik, Kassa (24) among hypertensive patients at the University of Gondar Comprehensive Specialized Hospital, Northwest Ethiopia. The knowledge section comprised 3 questions assessing the potential risk, warning signs, and risk factors related to knowledge about stroke. Questions included 1) knowledge of signs of stroke such as sudden onset of dizziness; sudden onset of headache; sudden onset of memory loss; sudden onset of half body weakness; sudden onset of loss of consciousness; sudden onset of double vision; and sudden onset of speech problems, and 2) knowledge of risk factors such as high blood pressure; smoking; diabetes mellitus; cardiac disease; obesity; high cholesterol; excessive alcohol intake; physical activity; and the presence of a family member having a stroke. Responses included: “Yes,” “No,” “I donť know;” with “Yes” responses coded as 1 and all other responses coded as 0. A composite score was generated based on the summation of all the correct responses. Thereafter, the median score (2.0) was used as a cut-off. Those who obtained a score equal to or above the median were considered to have a high knowledge of stroke. Physical activity was measured as exercising more than 3 times a week, at moderate intensity or more than 30 minutes each time, or engaging in moderate including walking and severe physical work.

### Data collection procedures and tools

Data was collected by Research Assistants (RAs) with minimum education qualification of a bachelor’s degree in Humanities, Statistics, Environmental or Public Health, or any other related discipline as well as prior experience in conducting face-to-face interviews. These RAs underwent a 3-day training to get acquainted with the study protocol, and the ethical issues about the study. A semi-structured questionnaire developed based on a thorough literature review of existing literature related to stroke prevalence and predictors in similar populations (25, 26) was used to obtain data. This questionnaire was administered through face-to-face interviews, and it elicited information on the: socio-demographic characteristics, patient-related factors, lifestyle factors, hypertension treatment-related factors, and health system factors.

The study questionnaire underwent validation by a team of stroke experts to ensure its accuracy and relevance (27). The experts reviewed the content to confirm that it covered all necessary aspects of stroke prevalence, predictors, and related factors, and provided feedback to refine the questions. Their validation ensured that the questionnaire was both reliable and valid for assessing stroke-related prevalence and risk factors in the target population. This questionnaire was developed in English and translated into Luganda, the most commonly spoken local language in GKMA. Pretesting of the questionnaires was done in public healthcare facilities in Mityana district before being used for the study to ensure clarity and suitability.

#### The data collection process;

The data collection team was divided into three teams of two, with each team in one district. One team of two research assistants collected data at each facility on the specific clinic days in the selected health facilities. At each health facility, permission was sought from the health facility in charge. With the in-charge’s support, eligible participants were identified, and if no eligible respondent was present at the time of the study, an appointment was made at a convenient time. If eligible respondents were present, informed consent was sought using a consent form. Data was collected from patients after they had been seen by the physician in the specialized hypertension clinics. However, recognizing that some participants may have limitations in verbal communication, I adapted this approach to ensure inclusivity. If a patient in the specialized hypertension clinic was unable to directly participate, their designated caregiver was interviewed instead. Informed consent was also sought from the caretaker ensuring their understanding of the study. The questionnaire was then administered at a place of convenience and privacy to the participant. This lasted 20–30 minutes per participant.

### Data management and analysis

Data were entered using the Kobo collect mobile application preloaded on Android-enabled mobile phones. RAs were required to upload the data daily to the cloud server for quality control purposes. Data was then downloaded into MS Excel 2016, cleaned, and analyzed using STATA 15.0 statistical software. Data cleaning involved the removal of unwanted or duplicate observations from the dataset (de-duplication), fixing structural errors such as typos, or incorrect capitalization, filtering unwanted outliers, handling missing data, and validation. Descriptive statistics such as mean and standard deviation were used to present continuous variables, while frequencies and proportions were used to present categorical data. Both bivariate and multivariable analyses were conducted to ascertain significant variables. In the bivariate analysis, a significance level of p < 0.1 was used, while in the multivariable analysis, the significance level was set at p < 0.05. The rationale for using a p-value cutoff of 0.1 in the bivariate analysis is to include potentially relevant variables that may have a weaker association with the outcome but could still be important in the context of the study (28, 29). This approach allows for a more comprehensive exploration of potential predictors before applying more stringent criteria in the multivariable analysis.

Since the outcome had a prevalence that exceeded 10%, a modified Poisson regression analysis with robust standard errors was used to report prevalence ratios. Multicollinearity effect was assessed; A correlation coefficient of ≥ 0.4 was considered high; if two variables exceeded this threshold, only one was retained in the model to avoid multicollinearity. To assess for interaction, the chunk test was used to compare a full model with interaction terms and a simplified model with only basic variables. Interaction terms were retained in the model if the test yielded a significant p-value < 0.05. Confounding was assessed on all variables that dropped out of the model using stepwise elimination, with assessment prioritized from the most significant to the least significant variable that dropped out. The results were presented in tables, graphs, and figures as appropriate.

### Quality control and assurance

The RAs were trained for 3 days to ensure that they fully understand the protocol and the data collection tools. Pretesting of the questionnaires was done in public healthcare facilities in Mityana district, before being used for the study to ensure clarity and suitability in assessing the stroke prevalence and associated factors. Mityana district was selected for the pretest due to its resemblance to the GKMA in key characteristics such as demographics, healthcare infrastructure, and socioeconomic status. During the pretest, we identified and addressed any issues related to question comprehension, response accuracy, and overall questionnaire flow. Feedback from the pretesting allowed us to refine the questions to ensure that the questions were understandable and relevant to the target population. Research assistants were supervised during the entire data collection period to ensure quality data. This supervision involved regular check-ins, on-the-spot reviews of collected data, and immediate feedback to address any issues or inconsistencies. Daily debrief meetings were held to identify challenges that arose during the data collection process and address them accordingly. The questionnaire embedded in the Kobo Collect app was programmed to ensure the completeness of the data entered. During programming, mandatory fields and skip patterns were inserted into the tool. Back translation was conducted to ensure that the meaning of the questions is not lost. After data collection, the data was cleaned in Excel 2016.

## Results

### Socio-demographics characteristics of respondents

Out of the 383 respondents, the majority 71.0% (272/383) were aged 60–69 years with a mean age being 66.8 ± 7.1 years. More than three-quarters of the respondents 80.9% (310/383) were female, 39.9% (153/383) were catholic, 42.8% (164/383) had a primary education level, and 71.5% (274/383) had dependents (Table 2).

### Patient and hypertension treatment-related factors of respondents

More than two-thirds, 69.2% (265/383) of the respondents were diagnosed with hypertension more than 2 years ago, and 98.4% (377/383) were taking any hypertension medication. Of those who were taking hypertension medication, 35.5% (136/377) were taking both medical and traditional medicines, 99.7% (376/377) had an oral antihypertension treatment, and 66.8% (252/377) had used the hypertension treatment for more than 2 years (Table 3).

### Lifestyle factors of respondents

About 31.9% (122/383) of the respondents always carried out physical exercise in a week, 94.8% (363/383) consumed carbohydrates, 5.2% (20/383) had ever smoked tobacco, and 42.0% (151/383) had ever drunk alcohol (Table 4).

### Health facility-related factors

More than three quarters 78.1% (299/383) of the respondents had visited the health facility 1 month ago, 36.0% (138/383) mentioned that antihypertensive medication was always available, and 69.5% (266/383) paid for antihypertensive medication. Only 1.0% (4/383) of the respondents were on health insurance, 93.0% (356/383) mentioned that the relationship between health workers and patients at the health facility was always good, and 46.7% (179/383) perceived the distance from their home to the health facility as being near (Table 5).

### Prevalence of stroke and stroke-related knowledge among respondents

The prevalence of stroke among respondents was 18.3% (70/383). The prevalence of stroke was 15.4% (42/272) in those aged 60–69, 22.5% (18/80) for those aged 70–79, and 32.2% (10/31) for those aged 80 and above. Of the respondents that had ever had a stroke, 10.4% (40/70) had it more than a year ago and 78.6% (55/383) still had any stroke-related symptoms. Regarding knowledge levels, 58.7% (225/383) of the respondents had high knowledge of stroke and 53.3% (204/383) had ever received stroke-related information (Table 6).

### Knowledge on the signs and symptoms of stroke among respondents

**Figure 1:**
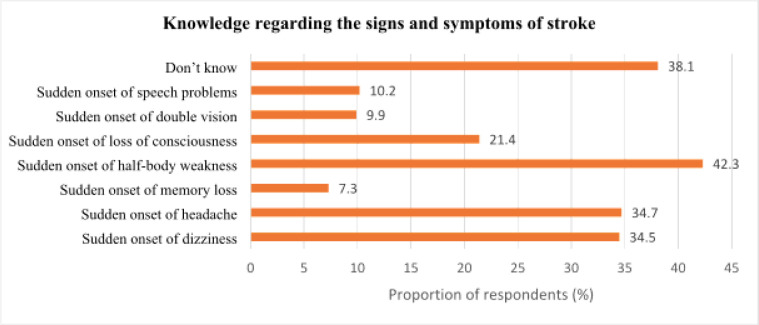
Knowledge regarding the signs and symptoms of stroke among respondents

Figure 1 shows the distribution of respondents' knowledge regarding the signs of stroke. The most frequently recognized sign was the sudden onset of half-body weakness (42.3%; 162/383), followed by sudden onset of headache (34.7%; 133/383), and sudden onset of dizziness (34.5%; 132/383). Other signs included sudden onset of loss of consciousness (21.4%; 82/383), sudden onset of speech problems (10.2%; 39/383), sudden onset of double vision (9.9%; 38/383), and sudden onset of memory loss (7.3%; 28/383), which were less frequently mentioned by respondents.

### Knowledge on stroke risk factors among respondents

**Figure 2:**
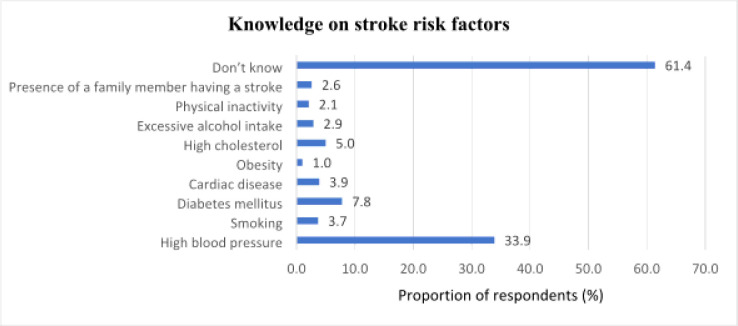
Knowledge on stroke risk factors among respondents

Figure 2 shows the distribution of respondents' knowledge regarding stroke risk factors. The most frequently recognized risk factor was high blood pressure (33.9%; 130/383). This was followed by diabetes mellitus (7.8%; 30/383) and high cholesterol (5.0%; 19/383). Other factors such as cardiac disease (3.9%; 15/383), smoking (3.7%; 14/383), and excessive alcohol intake (2.9%; 11/383) were mentioned less frequently. Physical inactivity (2.1%; 8/383), the presence of a family member having a stroke (2.6%; 10/383), and obesity (1.0%; 4/383) were the least recognized risk factors. Majority of respondents (61.4%; 235/383) indicated that they did not know any stroke risk factors.

### Predictors of stroke among older hypertensive patients attending public healthcare facilities in GKMA

After controlling for age, sex, religion, and education level, being 80 and above years, having 8–13 years of formal education (secondary education), having health insurance, having high knowledge of stroke, and receiving any stroke-related health information were significantly associated with stroke. Respondents aged 80 and above years (APR = 2.68, 95% CI:1.59–4.51) had a 168% higher prevalence of stroke as compared to those aged 60–69 years. Respondents with 8–13 years of formal education (secondary education) (APR = 0.37, 95% CI: 0.14–0.98) had a 63% lower prevalence of stroke as compared to those with no formal education. Respondents with health insurance (APR = 3.34, 95% CI: 1.19–9.37) had a 234% higher prevalence of stroke as compared to those without health insurance. The prevalence of stroke among respondents with high knowledge of stroke (APR = 24.72, 95% CI: 6.20–98.55) was 24.72 times higher than those with low knowledge. The prevalence of stroke among respondents who had ever received any stroke-related health information (APR = 1.78, 95% CI: 1.05–3.02) was 1.84 times higher than their counterparts (Table 7).

## Discussion

This study assessed the preventive practices, prevalence, and factors associated with stroke among older patients with hypertension attending public healthcare facilities in the GKMA, Uganda. The prevalence of stroke among these patients was found to be 18.3%. This prevalence is considered high when compared to the global average stroke prevalence among older adults, which typically ranges from 5–10% according to the WHO and other epidemiological studies (3, 30). This study’s stroke prevalence is comparable to a study in Ethiopia which found an 18.18% prevalence among adult patients with hypertension (31), but higher than studies conducted in Nigeria, Sidama, and Shanghai which reported incidences of 13.2%, 3.15%, and 10.8% respectively (25, 32). The current study identified an increasing prevalence of stroke with age among older patients with hypertension: 15.4% in those aged 60–69, 22.5% in those aged 70–79, and 32.2% in those aged 80 and above. This study's finding aligns with a study done by Fekadu, Chelkeba (33) in Jimma in Ethiopia. However, compared to findings from (25) in Shanghai, this study’s prevalence rates are notably higher across all age groups. This discrepancy can be attributed to several factors, including the facility-based nature of the current study compared to the population-based study, differences in healthcare infrastructure and access, variations in hypertension management and control, and the reliance on self-reported stroke diagnoses. The higher stroke prevalence in the current study indicates the urgent need for improved public health interventions to enhance hypertension management and stroke prevention strategies in urban areas.

Furthermore, this study revealed that the factors associated with stroke among older patients with hypertension were being aged 80 and above years, having 8–13 years of formal education (secondary education), having health insurance, high knowledge of stroke, and receiving stroke-related health information. The association between age and stroke prevalence is consistent with findings from other studies (25, 34), which also show a significant relationship between being aged 80 and above and stroke prevalence. Additionally, having a secondary education level was significantly associated with stroke prevalence, as observed in a study by (24) in Ethiopia. However, while some studies have identified health insurance as a protective factor against stroke (35–37), this study found a higher prevalence of stroke among insured individuals. It is important to note that this study did not directly assess whether health insurance might be contributing to longer life expectancy among the elderly, thereby potentially creating a perception of higher stroke prevalence in insured older patients with hypertension.

Increasing age was found to be significantly associated with prevalence of stroke. As people age, the long-term effects of hypertension contribute to significant vascular damage, increasing arterial stiffness and promoting atherosclerosis (38, 39). Both conditions worsen with age, raising the likelihood of cerebrovascular events such as stroke. Moreover, aging is associated with a decline in endothelial function and a rise in chronic inflammation, both of which exacerbate the vulnerability to stroke (40). In older patients, inadequate treatment acceptance in routine practice could contribute to the increased stroke risk (41–43). In this study, it was observed that having 8–13 years of formal education (secondary education) was associated with a lower prevalence of stroke, aligning with the understanding that education enhances health literacy, enabling individuals to better manage hypertension and make healthier lifestyle choices (44, 45). Educated individuals are more likely to engage in regular physical activity, maintain a balanced diet, and avoid risk factors such as smoking and excessive alcohol consumption (46, 47).

Interestingly, the study found that individuals with health insurance had a higher prevalence of stroke. While health insurance is generally considered a protective factor against severe health outcomes, some studies have suggested that insured individuals are more likely to seek medical attention and receive formal diagnoses for conditions, leading to higher reported prevalence rates. For instance, studies such as those by (41, 48) have highlighted the potential for insurance to improve access to healthcare, thus increasing the diagnosis of non-communicable diseases like stroke. However, in low-resource settings, insured individuals may still face challenges such as inadequate management of risk factors, which could contribute to higher stroke prevalence (49–51). This study supports these findings, indicating that having insurance does not always guarantee better health outcomes, especially in contexts where healthcare quality is uneven. Additionally, despite the assumption that those with health insurance might also have higher educational attainment, this study shows that insurance coverage remains generally low in the population, and having insurance does not necessarily correlate with secondary education in this context. Many people with insurance may not necessarily represent those with higher education levels. Thus, the higher prevalence of stroke in insured individuals may reflect increased healthcare engagement and diagnostic reporting rather than poorer health outcomes.

In addition, this study found that respondents with higher stroke knowledge were more likely to have experienced a stroke compared to those with lower knowledge. This result is not surprising, as individuals who have suffered a stroke are more likely to acquire detailed information about the condition through personal experience and medical care. Experiencing a stroke often increases awareness of symptoms, risk factors, and preventive measures, as patients receive health education during their diagnosis, treatment, and recovery process. This aligns with findings from Sirisha, Jala (52), which highlight that individuals with firsthand experience of a condition are more likely to have greater knowledge about it. However, the important implication of this finding lies in the need to increase stroke awareness among individuals who have not yet experienced a stroke. Those with lower stroke knowledge may be unaware of their risk factors or early symptoms, which could lead to delayed healthcare-seeking behavior and poorer outcomes. Public health efforts should therefore focus on sensitizing this group, particularly among older patients with hypertension, through targeted health education campaigns to ensure that they recognize stroke risks and engage in preventive measures early on.

Besides, this study found a higher prevalence of stroke among respondents who had received stroke-related health information. It is important to note that receiving stroke-related health information itself is not a risk factor for stroke. Rather, individuals who are at a higher risk for stroke or have already experienced a stroke are more likely to seek out or be provided with this information by healthcare professionals. Healthcare providers often prioritize stroke education for patients who have significant risk factors or have already had a stroke, as part of their ongoing care and management (53, 54). This can lead to a higher observed prevalence of stroke in this informed group, as these individuals are more engaged with the healthcare system and more likely to have their stroke risk factors identified and managed. The higher prevalence, therefore, reflects the correlation between increased stroke risk and the likelihood of receiving targeted health education, not causality between the two. The implication of this finding is that targeted stroke-related health education plays a key role in improving awareness among individuals at higher risk of stroke. However, it also highlights the need for broader public health efforts to ensure that stroke education reaches individuals who may not have experienced a stroke or exhibit visible risk factors but could still be at risk. Expanding stroke prevention campaigns to a wider population, including those who are not yet engaged with healthcare services or who do not perceive themselves as being at risk, can help in early detection and prevention.

## Strengths and Limitations

### Strengths

This study represents a pioneering effort to assess prevalence and factors associated with stroke among older patients with hypertension attending public healthcare facilities in GKMA, Uganda. Its novelty lies in providing a foundational understanding that can inform future research, policy-making, and targeted interventions within the region. A relatively large sample size of 383 respondents was recruited, enhancing the generalizability of the findings to similar populations. This robust sample size strengthens the validity of inferences drawn about the target population. Furthermore, the study employed established frameworks such as the WHO Social Determinants of Health, ensuring methodological rigor and grounding its insights in a well-recognized theoretical context.

### Limitations

This study has several limitations. Stroke measurement relied on self-reports, cross-referenced with medical records where possible, but the absence of a standardized tool to assess stroke may have affected the reliability of the prevalence estimates. Survivor bias may have influenced findings, as only individuals who survived a stroke were included. The analysis excluded some variables from the conceptual framework, potentially limiting comprehensiveness. Socio-economic status was not directly assessed, though proxies like education level and health insurance were included. Recall bias may have affected data accuracy, but efforts were made to focus on recent and objective details. As a facility-based study, stroke prevalence may be underestimated, excluding individuals with fatal strokes or those not seeking care at public facilities. Finally, findings are specific to public healthcare facility users in the GKMA and may not represent the broader population.

## Conclusions and recommendations

This study showed a high prevalence of stroke among older patients with hypertension. The factors associated with stroke were age (80 years and above), having 8–13 years of formal education (secondary education), possession of health insurance, high knowledge of stroke, and receipt of stroke-related health information. Notably, while higher knowledge of stroke and receipt of health information was associated with an increased likelihood of stroke, this relationship highlights that individuals at greater risk are more likely to seek out and receive education about stroke. Thus, rather than indicating that knowledge or education is a risk factor, it suggests that these factors serve as indicators of engagement with the healthcare system and recognition of risk. Based on the study findings, several recommendations are proposed to improve stroke prevention and management among older adults. Public health education programs should be enhanced to increase awareness of stroke prevention methods, particularly targeting older adults and individuals with hypertension. Expanding public education initiatives to underserved populations through community outreach is critical to reach those with limited knowledge or access to healthcare services. Age-specific hypertension management programs should be developed, focusing on older adults at higher risk, including those aged 80 and above. Access to healthcare services should be improved by creating affordable and comprehensive healthcare plans that cover preventive and management services for older adults. Male engagement in hypertension management should be encouraged through targeted outreach campaigns to address low health-seeking behavior among men. Individuals with stroke experience can be trained as peer educators in chronic care clinics to support stroke prevention and management efforts. Lastly, further research should be conducted to identify specific barriers to stroke prevention and develop tailored interventions to meet the needs of different populations.

## Figures and Tables

**Table 1:** Number of public healthcare facilities with NCD clinics in GKMA

District	No. of PHF with NCD clinics	High-volume NCD clinics selected
Kampala	6	2
Mukono	5	2
Wakiso	8	3
**Total**	**19**	**7**

**Table 2:** Socio-demographics characteristics of respondents

Variable	Attribute	Frequency	Percentage (%)
Age of the respondents (Mean = 66.8 ± 7.1	60–69 years	272	71.0
	70–79 years	80	20.9
	80 and above years	31	8.1
Sex of the respondent	Female	310	80.9
	Male	73	19.1
Religion	Anglican	84	21.9
	Catholic	153	39.9
	Muslim	43	11.2
	Pentecostal (born again)	87	22.7
	SDA	16	4.2
Education Level	No Formal education	152	39.7
	Primary (1–7 years)	164	42.8
	Secondary (8–13 years)	43	11.2
	Tertiary	24	6.3
Employment Status	Employed by someone	7	1.8
	Self-employed	127	33.2
	Unemployed	249	65.0
Marital Status	Divorced/ Separated	92	24.0
	Living together / Cohabiting	23	6.0
	Married	140	36.6
	Never Married/ Single	4	1.0
	Widowed	124	32.4
Dependents	No	109	28.5
	Yes	274	71.5
Number of dependents (n = 274)	1	14	5.1
	2–4	115	42.0
	5 and above	145	52.9

**Table 3:** Patient and hypertension treatment-related factors of respondents

Variable	Attribute	Frequency	Percentage (%)
Duration since diagnosed with hypertension	Below 1 year	22	5.7
	1–2 years	96	25.1
	Above 2 years	265	69.2
Currently taking any medications for hypertension	No	6	1.6
	Yes	377	98.4
Hypertension Medication taken (n = 377)	Both	136	35.5
	Medical	240	62.7
	Traditional or alternative medicines	1	0.3
Frequency of monitoring blood pressure	Always	267	69.7
	Never	3	0.8
	Sometimes	113	29.5
Route of administration for your hypertension treatment (n = 377)	Oral	376	99.7
	Injectable	1	0.3
If oral, number of tablets taken per day (n = 376)	1	170	45.2
	2–4	196	52.1
	5 and more	10	2.7
Period on hypertension medication (n = 377)	Below 1 year	37	9.8
	1–2 years	88	23.3
	Above 2 years	252	66.8
Person/entity paying for the medications	Self	243	63.4
	Relative	74	19.3
	Health facility provides free	145	37.9
	Insurance	3	0.8
Challenges faced while taking antihypertensive medication*	Drug is expensive	240	62.7
	Do not understand prescription	4	1.0
	Too many tablets	3	0.8
	The drug is hard to swallow	18	4.7
	Drugs are not always available	95	24.8
	Normally forget to take it because I am always busy	18	4.7

* Multiple response

**Table 4:** Lifestyle factors of respondents

Variable	Attribute	Frequency	Percentage (%)
Number of days physically active for a total of at least one hour per day bin the past seven days	0 days	109	28.4
	1 day	31	8.1
	2 days	72	18.8
	3 days	41	10.7
	4 days	26	6.8
	5 days	11	2.9
	6 days	11	2.9
	7 days	82	21.4
Frequency of carrying out physical exercise in a week	Always	122	31.9
	Never	94	24.5
	Rarely	42	11.0
	Sometimes	125	32.6
Food commonly consumed*	Carbohydrates	363	94.8
	Proteins	291	76.0
	Fruit	230	60.1
	Vegetables	330	86.2
Ever smoked tobacco	No	363	94.8
	Yes	20	5.2
Still smokes tobacco (n = 20)	No	16	80.0
	Yes	4	20.0
Ever drunk alcohol	No	222	58.0
	Yes	161	42.0
Still takes alcohol (n = 161)	No	121	75.2
	Yes	40	24.8

* Multiple response

**Table 5:** Health facility-related factors

Variable	Attribute	Frequency	Percentage (%)
Last time visited the health facility (Months)	1 month	299	78.1
	More than 1 month	84	21.9
Period the health worker recommended the patient report back to the facility (Months)	1 month	304	79.4
	More than 1 month	79	20.6
Perception about the availability of antihypertensive medication in the health facility	Always available	138	36.0
	Available but expensive	125	32.7
	Not always available	120	31.3
Pays for antihypertensive medication at this facility	No	117	30.5
	Yes	266	69.5
Perception about the cost of medication at this facility	Affordable	141	36.8
	Don’t Know	51	13.3
	Expensive	191	49.9
On health insurance	No	379	99.0
	Yes	4	1.0
Perception about the relationship between health workers and patients in this health facility	Always good	356	93.0
	Not good	1	0.3
	Sometimes good	26	6.8
Time taken when traveling from home to the health facility	30 minutes and below	195	50.9
	Above 30 minutes	188	49.1
Perception about the distance from your home to the health facility	Can’t say	3	0.8
	Far	201	52.5
	Near	179	46.7
Challenges faced while accessing this health facility*	Transport is expensive	218	56.9
	Traffic congestion on the road	46	12.0
	Motion sickness while traveling	44	11.5
	I cannot come on my own(disabled)	13	3.4
	None	134	35.0

* Multiple response

**Table 6:** Prevalence of stroke and stroke-related knowledge among respondents

Variable	Attribute	Frequency	Percentage (%)
Ever gotten a stroke	No	313	81.7
	Yes	70	18.3
Duration since diagnosed with stroke (n = 70)	Below 1 year	30	7.8
	1 year and above	40	10.4
Still has any stroke-related symptoms (n = 70)	No	15	21.4
	Yes	55	78.6
Ever received any stroke-related health information	No	168	43.9
	Not sure	11	2.9
	Yes	204	53.3
Source of the stroke-related health information*	VHT	8	2.1
	Health care provider	183	47.8
	Internet/Media	11	2.9
Perceived risk of experiencing a stroke	No	281	73.4
	Yes	102	26.6
	Obesity	4	1.0
	High cholesterol	19	5.0
	Excessive alcohol intake	11	2.9
	Physical inactivity	8	2.1
	Presence of a family member having a stroke	10	2.6
	Don’t know	235	61.4

* Multiple response

**Table 7:** Predictors of stroke among older hypertensive patients attending public healthcare facilities in GKMA

Variable	Attribute	Ever had a stroke		Crude PR (95% CI)	P-values	Adjusted PR (95% CI)	P-values
		Yes (n = 70)	No (n = 313)				
Age of the respondent	60–69	42 (15.4)	230 (84.6)	1		1	
	70–79	18 (22.5)	62 (77.5)	1.46 (0.89–2.39)	0.135	1.28 (0.82–2.01)	0.278
	80 and above	10 (32.3)	21 (67.7)	2.09 (1.17–3.74)	0.013	2.68 (1.59–4.51)	< 0.001*
Sex of the respondent	Female	56 (18.1)	254 (81.9)	1		1	
	Male	14 (19.2)	59 (80.8)	1.06 (0.63–1.80)	0.824	1.34 (0.81–2.21)	0.246
Religion	Anglican	16 (19.0)	68 (81.0)	1		1	
	Catholic	24 (15.7)	129 (84.3)	0.82 (0.46–1.46)	0.508	0.60 (0.35–1.03)	0.064
	Muslim	9 (20.9)	34 (79.1)	1.10 (0.53–2.28)	0.800	0.86 (0.45–1.67)	0.663
	Pentecostal (born again)	15 (17.2)	72 (82.8)	0.9 (0.48–1.71)	0.760	0.65 (0.35–1.20)	0.171
	SDA	6 (37.5)	10 (62.5)	1.97 (0.91–4.26)	0.085	1.11 (0.55–2.28)	0.763
Education Level	No Formal education	25 (16.4)	127 (83.6)	1		1	
	Primary	36 (22.0)	128 (78.0)	1.33 (0.84–2.11)	0.219	1.14 (0.74–1.75)	0.561
	Secondary	4 (9.3)	39 (90.7)	0.56 (0.21–1.54)	0.264	0.37 (0.14–0.98)	0.046*
	Tertiary	5 (20.8)	19 (79.2)	1.27 (0.54–2.99)	0.590	0.90 (0.41–1.96)	0.788
Employment Status	Employed by someone	2 (28.6)	5 (71.4)	1			
	Self-employed	19 (15.0)	108 (85.0)	0.52 (0.15–1.82)	0.308		
	Unemployed	49 (19.7)	200 (80.3)	0.69 (0.21–2.28)	0.542		
Has dependents	No	19 (17.4)	90 (82.6)	1			
	Yes	51 (18.6)	223 (81.4)	1.07 (0.66–1.72)	0.788		
Duration since diagnosed with hypertension	Below 1 year	4 (18.2)	18 (81.8)	1			
	1–2 years	15 (15.6)	81 (84.4)	0.86 (0.31–2.34)	0.767		
	Above 2 years	51 (19.2)	214 (80.8)	1.06 (0.42–2.66)	0.904		
Frequency of monitoring blood pressure	Always	47 (17.6)	220 (82.4)	1			
	Never	1 (33.3)	2 (66.7)	1.89 (0.37–9.60)	0.441		
	Sometimes	22 (19.5)	91 (80.5)	1.11 (0.70–1.75)	0.665		
Frequency of carrying out physical exercise in a week	Always	21 (17.2)	101 (82.8)	1			
	Never	15 (16.0)	79 (84.0)	0.93 (0.50–1.70)	0.807		
	Rarely	9 (21.4)	33 (78.6)	1.24 (0.62–2.50)	0.539		
	Sometimes	25 (20.0)	100 (80.0)	1.16 (0.69–1.96)	0.575		
Ever smoked tobacco	No	66 (18.2)	297 (81.8)	1			
	Yes	4 (20.0)	16 (80.0)	1.10 (0.44–2.72)	0.836		
Ever drunk alcohol	No	39 (17.6)	183 (82.4)	1			
	Yes	31 (19.3)	130 (80.7)	1.10 (0.71–1.68)	0.673		
Perception about the availability of antihypertensive medication in the health facility	Always available	20 (14.5)	118 (85.5)	1			
	Available but expensive	26 (20.8)	99 (79.2)	1.43 (0.84–2.44)	0.182		
	Not always available	24 (20.0)	96 (80.0)	1.38 (0.80–2.37)	0.244		
Pays for antihypertensive medication at this facility	No	24 (20.5)	93 (79.5)	1			
	Yes	46 (17.3)	220 (82.7)	0.84 (0.54–1.31)	0.451		
Perception about the cost of medication at this facility	Affordable	23 (16.3)	118 (83.7)	1			
	Don’t Know	10 (19.6)	41 (80.4)	1.20 (0.61–2.35)	0.591		
	Expensive	37 (19.4)	154 (80.6)	1.19 (0.74–1.91)	0.477		
Health insurance	No	68 (17.9)	311 (82.1)	1		1	
	Yes	2 (50.0)	2 (50.0)	2.79 (1.02–7.61)	0.046	3.34 (1.19–9.37)	0.022*
Perception about the relationship between health workers and patients in this health facility	Always good	62 (17.4)	294 (82.6)	1		1	
	Not good/sometimes good	8 (29.6)	19 (70.4)	1.70 (0.91–3.18)	0.095	1.66 (0.99–2.78)	0.053
Time taken when traveling from home to the health facility	30 minutes and below	37 (19.0)	158 (81.0)	1			
	Above 30 minutes	33 (17.6)	155 (82.4)	0.92 (0.60–1.41)	0.720		
Knowledge of stroke	Low	2 (13)	156 (98.7)	1		1	
	High	68 (30.2)	157 (69.8)	23.87 (5.93–96.16)	< 0.001	24.72 (6.20–98.55)	< 0.001*
Ever received any stroke-related health information	No	19 (11.3)	149 (88.7)	1		1	
	Not sure	1 (9.1)	10 (90.9)	0.80 (0.12–5.47)	0.823	1.57 (0.31–7.81)	0.584
	Yes	50 (24.5)	154 (75.5)	2.17 (1.33–3.53)	0.002	1.78 (1.05–3.02)	0.031*

## Data Availability

The datasets used and/or analysed during the current study are available from the corresponding author upon reasonable request.

## Abbreviations

CDC: Centre for Disease Control
DALYs: Disability Adjusted Life Years
GKMA: Greater Kampala Metropolitan Area
GOU: Government of Uganda
MoH: Ministry of Health
NCD: Non-Communicable Disease
SDGs: Sustainable Development Goals
SSA: Sub- Saharan Africa
WHO: World Health Organization
